# Primary Hyperparathyroidism in Sickle Cell Disease: An Unknown Complication of the Disease in Adulthood

**DOI:** 10.3390/jcm9020308

**Published:** 2020-01-22

**Authors:** Elsa Denoix, Charlène Bomahou, Lorraine Clavier, Jean-Antoine Ribeil, François Lionnet, Pablo Bartolucci, Marie Courbebaisse, Jacques Pouchot, Jean-Benoît Arlet

**Affiliations:** 1 Internal Medicine Department, Sickle Cell Referral Center, Georges Pompidou European Hospital, Assistance Publique-Hôpitaux de Paris, 20 rue Leblanc, 75015 Paris, France; elsa.denoix@gmail.com (E.D.); charleneboma2@hotmail.fr (C.B.); jacques.pouchot@aphp.fr (J.P.); 2 Faculté de Médecine Paris Descartes, Université de Paris, 75015 Paris, France; jaribeil@gmail.com (J.-A.R.); marie.courbebaisse@aphp.fr (M.C.); 3 Service de Diabétologie-Endocrinologie, Hôpitaux Universitaires Henri Mondor-Albert Chenevier, Assistance Publique-Hôpitaux de Paris, Université Paris-Est Créteil, 94010 Creteil, France; lorraine.clavier@aphp.fr; 4 Laboratory of Excellence GR-Ex, 75015 Paris, France; 5 Biotherapy Department, Sickle Cell Referral Center, Necker Children’s Hospital, Assistance Publique-Hôpitaux de Paris, 75015 Paris, France; 6 Service de Médecine Interne, Sickle Cell Referral Center, Hôpital Tenon (AP-HP), 4, rue de la Chine, 75020 Paris, France; francois.lionnet@aphp.fr; 7 Sickle Cell Referral Center, Hôpitaux Universitaires Henri Mondor-Albert Chenevier, Assistance Publique-Hôpitaux de Paris, Université Paris-Est Créteil, 94010 Creteil, France; pablo.bartolucci@aphp.fr; 8 Institut Mondor de Recherche Biomédicale (IMRB), Institut National de la Santé et de le Recherche Médicale (INSERM) U955, DHU A-TVB, F-94010 Créteil, France; 9 Physiology Department, Georges Pompidou European Hospital, Assistance Publique-Hôpitaux de Paris, 75015 Paris, France

**Keywords:** primary hyperparathyroidism, adenoma, hypercalcemia, sickle cell disease, osteoporosis

## Abstract

Primary hyperparathyroidism (pHPT) is the third most common endocrine disorder and usually affects patients between 60 and 70 years of age. To our knowledge, this condition has never been studied in young patients with sickle cell disease (SCD). Our objective was to describe the clinical and biological characteristics of pHPT in adult patients with SCD and its management. We conducted a retrospective study that included SCD patients who were diagnosed with pHPT in four SCD referral centers. pHPT was defined by the presence of elevated serum calcium levels with inappropriate normal or increased parathyroid hormone (PTH) serum levels or histopathological evidence of parathyroid adenoma or hyperplasia. Patients with severe renal impairment (GFR <30 mL/min) were excluded. Twenty-eight patients (18 women, 64%; 22 homozygous genotype, 79%) were included. The median age at pHPT diagnosis was 41 years (interquartile range –IQR- 31.5–49.5). The median serum calcium and PTH concentration were, respectively, 2.62 mmol/L (IQR 2.60–2.78) and 105 pg/mL (IQR 69–137). Bone mineral density (BMD) revealed very low BMD (≤−2.5 SD) in 44% of patients explored (vs. 12.5% among 32 SCD patients matched for SCD genotype, sex, age, and BMI, *p* = 0.03). Fourteen patients (50%) received surgical treatment, which was successful in all cases, but four of these patients (29%) presented with pHPT recurrence after a median time of 6.5 years. Three of these patients underwent a second cervical surgery that confirmed the presence of a new parathyroid adenoma. These results suggest that SCD is a condition associated with pHPT in young subjects. SCD patients with pHPT have a high risk of very low BMD. A diagnosis of pHPT should be suspected in the presence of mild hypercalcemia or low BMD in SCD patients.

## 1. Introduction

Primary hyperparathyroidism (pHPT) is a common endocrine disorder. The peak incidence of pHPT is between 60 and 70 years of age. pHPT is more common in women, with a sex ratio of approximately 3:1 in patients over 50 years of age [1]. In most cases, the disease is subclinical and is often detected by an incidental finding of hypercalcemia. Single parathyroid gland adenoma is the most common cause (80%), parathyroid hyperplasia occurs in nearly 20% of cases, and parathyroid carcinoma is rare (~1%). The pHPT incidence depends on the populations studied. It has been estimated to be 21.6 per 100,000 person-years in the United States [1] and less than 6 per 100,000 person-years in Europe [2,3]. Some reports suggest that pHPT could be more common and symptomatic in black people [4,5].

Although pHPT mostly presents sporadically, some mutations have been linked to pHPT in young subjects, including multiple endocrine neoplasia (MEN) syndrome, either type 1 (caused by a mutation in *MEN1*) or type 2a (caused by a mutation in *RET*), and hyperparathyroidism-jaw tumor syndrome (caused by a mutation in *HRPT2*). In patients under 45 years of age presenting with pHPT, these mutations were identified in 23.5%, and the *MEN1* mutation was the most common [6]. Therefore, according to the international guidelines from 2012, *MEN1* mutation screening is recommended for patients with pHPT under 30 years of age [7]. A differential diagnosis of pHPT is familial benign hypocalciuric hypercalcemia, which is caused by a mutation of the *CaSR* gene and requires no specific treatment. 

Sickle cell disease (SCD) is an inherited disease that is characterized by misshapen red blood cells and predominantly affects people of African ancestry. In certain conditions, abnormal hemoblobin S (HbS) polymerizes, transforming the normally flexible red cell shape into an elongated rigid form. These sickled red blood cells have greatly reduced deformability, leading to occlusion of the microvasculature. Repeated ischemia–reperfusion episodes are responsible for many of the acute complications (including painful bone vaso-occlusive crisis, acute chest syndrome, priapism, stroke, and splenic sequestration) and chronic organ damage (retinopathy, avascular osteonecrosis, leg ulcers, nephropathy, pulmonary hypertension, etc.). Thus, the pathology of SCD can be directly attributed to HbS polymerization and vaso-occlusion, as well as to secondary effects of chronic hemolysis and complex inflammatory processes [8].

SCD is not considered a condition associated with pHPT, and, to our knowledge, only two case reports involving SCD patients with pHPT have been previously reported: a 17-year-old girl [9] and a 59-year-old woman [10]. They both had a parathyroid adenoma. However, in a previous study exploring vitamin D deficiency and its consequences in a prospective cohort of 56 consecutive adult SCD patients (mean age 29.8 ± 9.5 years), we identified pHPT in three patients [11]. Such a prevalence (5%) was very unusual in these young patients since the pHPT prevalence in black Americans between 40 and 49 years of age has been estimated at 0.1% [4]. 

Therefore, the aim of the present study was to better delineate the clinical and biological characteristics and review management of pHTP in adult patients with SCD. 

## 2. Methods

Cases of pHPT in adults (age >18 years) with SCD were retrospectively reviewed in four SCD referral centers in the Paris area. pHPT was defined by an elevated serum calcium level or ionized calcium level with an inappropriate intact parathyroid hormone (PTH) level (normal or elevated) or histopathological proof of parathyroid adenoma or hyperplasia. To rule out tertiary hyperparathyroidism, patients with an estimated glomerular filtration rate (eGFR) <30 mL/min/1.73 m^2^ were excluded. The eGFR was calculated using the Chronic Kidney Disease Epidemiology Collaboration (CKD-EPI) formula without considering a correction for ethnicity, which is the best method for estimating GFR in SCD [12]. Three patients were already included in a previous study, but their clinical and biological data and pHPT management strategies were not detailed [11]. 

A comprehensive review of the medical record of each patient was performed. The collected clinical information included age, sex, SCD genotype, parents’ country of origin, body mass index (BMI), and treatments for SCD and pHPT. To assess the possible presence of classic manifestations of pHPT, we recorded past medical histories of gastric ulcer and kidney stone. Lithium and thiazide diuretic prescriptions were specifically recorded because these drugs can interfere with the serum calcium and PTH levels. The collected biological data included serum levels of total calcium, ionized calcium, phosphate, PTH, 25(OH) vitamin D, hemoglobin level, and reticulocyte count at the time of pHPT diagnosis. The calcium, phosphate, and PTH levels were analyzed according to the laboratory cut-offs of each hospital (Supplemental Table S1). PTH was measured using a second-generation assay: whole PTH (Roche Diagnostics GmbH ®, Mannheim, Germany), on Elecsys 2010® and Cobas e411®. This assay, being a second-generation assay, detects PTH_1-84_ and PTH_7-84_. All SCD patients with pPTH followed in two of the four centers of the study (Pompidou and Necker hospital) underwent an oral calcium load test in an expert specialized physiology department. The protocol and performance characteristics of this test have been previously described [13]. The diagnosis of primary hyperparathyroidism was established when, during the test, serum ionized calcium concentration increased to supranormal values, and only a minimal reduction in serum PTH concentration was seen.

The results of neck ultrasonography (US) and Tc99m-sestamibi scintigraphy were collected when available. Clinical and biological outcomes after parathyroid gland surgery and parathyroid histopathology were recorded.

To assess bone turnover, serum bone formation and bone resorption markers (osteocalcin and C-terminal telopeptide of type I collagen (CTX), respectively) were collected when available. Bone mineral density (BMD) results were also collected at three sites: the lumbar spine (L1–L4), hip, and distal radius. Low BMD was defined as −2.5 < T-score < −1 SD and very low BMD (osteoporosis for menopausal women or men older than 50 years old) was defined as a T-score ≤ −2.5 SD in the lumbar spine or in the hip according to the WHO classification. BMD was measured using dual X-ray absorptiometry with the device of each center. We compared the BMD values and the low BMD prevalence to those of a control group of SCD patients who were matched for sex, age, SCD genotype, and BMI. This control group was generated from 151 consecutive SCD patients followed in one of the centers. These patients underwent BMD assessments at a steady state of their disease. 

The results of mutations related to pHPT (*MEN 1*, *RET*, and *HRPT2*) or to familial benign hypocalciuric hypercalcemia (*CaSR*) were collected when available.

### Statistical Analysis

Descriptive data are presented as the percentage or the median (range or IQR). The clinical, biological, and demographic parameters of the patients who underwent parathyroid gland surgery were compared to those of patients who did not receive surgery. Biological parameters before and after surgery in the surgical group were compared. The low BMD prevalence and T-score values in our patients with BMD assessments were compared to those of the matched control group defined above. Fisher’s exact test was used to compare percentages. The Mann–Whitney test was used to compare quantitative variables between two groups. *p* values less than 0.05 were considered significant. All analyses were performed using R version 3.3.1.

## 3. Results

### 3.1. Clinical Characteristics 

Thirty-five patients with hyperparathyroidism were initially identified, but seven were excluded, including one with secondary hyperparathyroidism and six with severe renal impairment. 

Twenty-eight patients (18 women and 10 men) with a median age at the time of pHPT diagnosis of 41 years (IQR: 31.5–49.2) were included (Table 1). All patients were identified between 2005 and 2016, excepted two patients who were diagnosed in 1999. The SCD genotype was SS in 22 (79%) patients, SC in four patients, and Sβ^+^thalassemia in two patients. The patients originated from Sub-Saharan Africa (*n* = 23, 82%), the Caribbean (*n* = 4), and North Africa (*n* = 1). No patient was treated with lithium or a thiazide diuretic. All patients were asymptomatic at the time of pHPT diagnosis. Two patients had a past medical history of gastric ulcer, and three had a history of kidney stones. There was no reported history of prior fragility fractures.

### 3.2. Biological Characteristics 

pHPT was diagnosed following an incidental finding of hypercalcemia in 26 patients (93%). We were unable to identify retrospectively the reason for parathyroid metabolism exploration in the remaining two patients, who may have had normocalcemic hyperparathyroidism. Indeed, both had normal serum albumin-adjusted total calcium serum levels and high serum levels of PTH (106 and 102 pg/mmol, respectively (normal range: 15–65)) without vitamin D deficiency. These two patients had abnormal parathyroid imaging and histopathological confirmation of pHPT after parathyroid gland surgery (parathyroid adenoma in one and parathyroid hyperplasia in the other).

Hypercalcemia was generally mild (the median total calcemia was 2.62 mmol/L (IQR: 2.6–2.78)) (Table 2). Only four patients (14.3%) had total calcemia ≥3 mmol/L. PTH was high in all patients, except in six (21.4%) who had hypercalcemia associated with an inappropriate normal PTH level. 

Eight patients (29%) had a low serum level of phosphate. The median 25-OH vitamin D concentration was 25.9 ng/mL (IQR: 12.7–48.2). Twelve patients (50%) had vitamin D insufficiency (10 ≤ 25(OH)D < 30 ng/mL). Three patients were vitamin-D-deficient (<10 ng/mL) but had evidence of pHPT with total hypercalcemia, and two of these patients had histopathological confirmation after parathyroid gland surgery. Twelve patients underwent an oral calcium load test in an expert specialized physiology department that confirmed inadequate suppression of PTH in all tested patients. The fasting urinary calcium/creatinine ratio was measured in samples of first morning urine for these 12 patients: three had elevated urinary calcium (none with a history of kidney stone) and two had low urinary calcium, but none of these subjects had a *CasR* mutation.

### 3.3. Bone Mineral Density 

Dual X-ray absorptiometry was performed in 16 of the 28 patients (median age: 40 years (34–49)) (Table 2). Seven patients (44%) who were explored had very low BMD (lumbar spine *n* = 5, lumbar spine and femoral neck *n* = 2), two had low BMD, and seven (44%) had normal or high BMD. Prevalence of patients with Z-score < −2 was 44% (seven patients). The median age of the patients with very low BMD was 45 years (range: 25–67), which was not significantly different from that of the patients with normal BMD. The prevalence of very low BMD was significantly higher in the SCD patients with pHPT compared to the matched control group of adult SCD patients without pHPT (44% vs. 12.5%, *p* = 0.03), indicating that pHPT could have an additive deleterious skeletal effect in a subset of adult SCD patients (Table 3). 

### 3.4. Parathyroid Imaging

**Figure 1:**
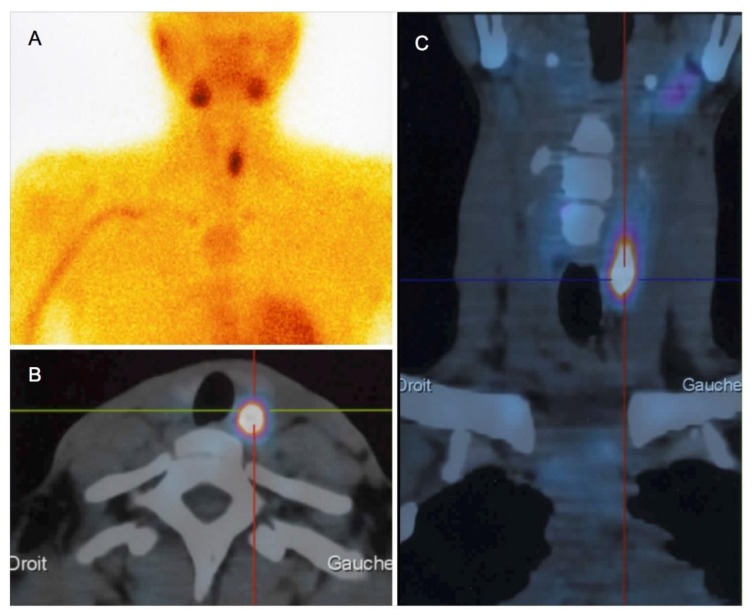
Tc99m-sestamibi scintigraphy (**A**) polled to computed tomography scans (axial (**B**) and coronal sections (**C**)) in a 30-year-old woman with pHPT and a 26-mm adenoma.

Twenty-three patients (82%) had parathyroid imaging: 22 had both neck US and Tc99m-sestamibi scintigraphy, and one had Tc99m-sestamibi scintigraphy only. Neck US findings were abnormal in 21 patients (95%): 19 had evidence of parathyroid adenoma (90%), and parathyroid hyperplasia was identified in the remaining two patients (10%). The median adenoma size (longest side on US) was 14.4 mm (range: 6–26 mm). Twenty-three patients had Tc99m-sestamibi scintigraphy, with focal fixation found in 20 patients (87%) (Figure 1) and normal results in the remaining three patients. Both imaging modalities were discordant in only three patients (3/22, 13.6%), and none of these patients were surgical candidates. 

### 3.5. Genetic Abnormalities

None of the patients had a familial history of pHPT. Gene mutations associated with pHPT were screened in 15 patients (54%), mainly in those who were diagnosed after 2010. The median age of these patients was 42 years old (IQR: 33.5–47). Thirteen of the 22 patients (59%) who were under 50 years of age were screened (*MEN 1* in 11, *RET* in one, and *HRPT2* in four), as recommended in the 2016 French guidelines, and no mutations were identified. Eleven patients were screened for a *CaSR* mutation, and all were negative, including the two patients with hypocalciuria (see above).

Among the 14 patients without surgical parathyroidectomy, seven were screened for CasR mutation. In the remainders, five had evidence of parathyroid adenoma on parathyroid imaging. In the two remaining patients, one had very elevated calcium rate (2.97 mmol/L), which is not in favor of hypocalciuric hypercalcemia. We cannot rule out hypocalciuric hypercalcemia for sure for the last one. Unfortunately, fasting urinary calcium/creatinine ratio and *CaSR* mutation were not screened for this patient.

### 3.6. pHPT Treatment 

Vitamin D supplementation was given to 23 patients (82%). 

Fourteen (50%) patients received surgical treatment. Cinacalcet, a calcimimetic, oral medical treatment of pHTP, was given in only three of the 14 non-operative patients during the follow-up. The median time between pHPT diagnosis and surgery was 4.25 years (range: 0.1–10). Cinacalcet was prescribed in 5/14 patients before surgery. Prophylactic exchange transfusions were administered to eight patients before surgery (57%) to prevent a post-surgical vaso-occlusive crisis. Histopathology identified 11 parathyroid adenomas and three cases of parathyroid hyperplasia. One patient had two parathyroid adenomas. Two of the three patients with parathyroid hyperplasia were screened for *MEN1* mutation and were negative. Surgical treatment was successful in all patients (normalization of serum calcium and PTH levels (Table 4)). Unfavorable postoperative outcomes occurred in three patients (21.5%): one had transient symptomatic hypocalcemia, one had laryngeal nerve paralysis, and one experienced an acute bone vaso-occlusive crisis (this patient did not receive prophylactic transfusion). No acute chest syndrome was observed as a postsurgical event. 

There was no clinical or biological difference between the surgical patients and the others at the time of pHTP diagnosis (Table 5). Thus, there was no apparent reason some patients had surgery and some did not. Nevertheless, a non-significant trend toward a higher prevalence of low BMD can be noted in the surgery group (67% vs. 29%).

### 3.7. Follow-Up

The median follow-up time after pHPT diagnosis was 4.55 years (IQR: 3.25–7). No specific therapy for pHPT was done in patients who did not undergo parathyroidectomy, except three patients treated by cinacalcet. None of them developed a severe hypercalcemia or fractures. Two patients died from conditions unrelated to pHPT: a 43-year-old man died from stercoral peritonitis five years after pHPT diagnosis (he did not undergo parathyroid surgery), and a 55-year-old woman died from status epilepticus 30 months after parathyroid gland surgery. 

The median follow-up time after cervical surgery was 2.25 years (IQR: 1.62–3.0). Four patients (28.6%) had pHPT recurrence after the first surgery, and three of them underwent a second surgery that demonstrated a new parathyroid adenoma. The median time to recurrence was 6.2 years (range: 4–8). *MEN1* and *RET* mutations were screened in one patient with pHPT recurrence, and the results were negative.

## 4. Discussion

### 4.1. General Considerations

To our knowledge, this is the first study to provide data on pHPT in SCD patients. 

The median age at pHPT diagnosis was 41 years, which is quite old compared to our adult SCD population. Indeed, the median age of the SCD patients was 33 years (IQR: 25–42) in a recent prospective study conducted in a cohort of 267 adults at the same centers [14]. Moreover, the median age of death in France was 36 years (IQR 23–50) in the 2003–2010 period [15]. However, our pHPT patients were significantly younger than those reported in a large series of pHPT cases: the mean age was 67 ± 14 years in 2709 European patients [16] and 64.2 ± 12.6 years in 1191 Black patients [4]. Notably, seven patients (25%) in our series were under 30 years of age. 

The retrospective design of our study did not allow us to correctly assess a prevalence of pHPT in SCD patients, since all of our approximately 2800 adult SCD patients followed during this period did not benefit from a systematic screening or a specific attention for mild hypercalcemia. The dosage of the ionized calcemia, more sensitive for the detection of pHTP, was also not performed routinely in SCD patients. The high prevalence of parathyroid adenoma recurrence after surgery despite the short follow-up (29% vs. 2.6% in a large study including 1402 patients with pHPT [17] and 10.7% in a more recent study with >3 years follow-up [18]) could reflect a pathophysiological link between pHPT and SCD and provides evidence that it is not a fortuitous association, despite a possible detection bias due to the regular medical follow-ups that SCD patients should undergo. Nevertheless, pHPT has not been reported to be more prevalent in other chronic disorders involving young patients with regular follow-ups, such as cystic fibrosis or type 1 diabetes mellitus. 

Except for age, the clinical, biological, and imaging features of pHPT in patients with SCD seem similar to those of other patient populations with this condition. Indeed, pHPT was mostly clinically asymptomatic. Hypercalcemia was usually moderate (only four patients presented with a calcium level ≥3 mmol/L). As expected, a low serum phosphate level was not uncommon and was found in eight (29%) of our patients [19]. Adenoma size was similar to that reported in other studies in non-SCD patients (14.4 mm vs. 19 mm in a recent study including 32 patients with parathyroid adenomas [20]). 

### 4.2. Bone Involvement

Severe bone disease in pHPT (osteitis fibrosa cystica) has become exceptional. However, low BMD, especially at distal radius and femoral neck sites, due to the catabolic effects of PTH in cortical bone, with an increased risk of fracture, is a common complication of pHPT and is the primary reason for parathyroid gland surgery [21]. Low BMD (<−1 SD) is also highly prevalent in young adult SCD patients, especially at the lumbar spine site, varying from 36% to 72% among studies, with the “osteoporosis” prevalence varying from 8% to 30% [22,23,24,25]. Of note, we used the T-score in our study to permit a comparison with published data about BMD in adult SCD patients and because 4/16 patients with BMD were older than 50 years. A Z-score and a cut-off < −2 should be preferably used in young patients. Increased hematopoiesis, an inflammatory state, and chronic vitamin D deficiency may be involved in low bone density [24,25]. 

In the present study, even if BMD was not performed for all patients with pHPT (as it is recommended), the prevalence of very low BMD was high among such young patients (44%). The osteoporosis prevalence in patients with pHTP is similar to that in the general population, but these patients were significantly older (48.4% of the 461 patients in a recent retrospective study, the prevalence increasing with age) [26]. It was also significantly higher than that in the control SCD patient group. Moreover, the distal radius T-scores, which were only available for five patients, were very low (median −2.6 (−5.1; −1)), thus confirming that pHPT has an additive deleterious bone effect in a subset of these patients. 

### 4.3. Treatment

Age younger than 50 years is a surgical indication in the management of pHPT according to the International Workshop on asymptomatic pHPT [27]. Surprisingly, only 10 of the 22 patients under 50 years of age in our series underwent surgical treatment, which was delayed after pHPT diagnosis. Patient and physician concerns regarding the postoperative risk of vaso-occlusive crisis or other more serious complications of SCD could explain this low surgical rate. Even if no threatening hypercalcemia or fracture were noted in SCD patients without surgical treatment during more than four years of follow-up, we did not consider it a sufficient reason to not recommend a parathyroidectomy to cure a pHTP in a SCD patient. Indeed, the benefit/risk and cost-effectiveness of parathyroidectomy on the long-term bone and calcium metabolism are well evidenced in the general population, with asymptomatic disease, particularly in young adults, even if only approximately 30% of these patients are treated operatively [28]. Moreover, our results provide reassurance regarding parathyroid surgery in this patient population because we observed no serious post-surgical complications, particularly no acute chest syndrome and no related death, even though 43% of the operated patients did not receive a prophylactic transfusion. Moreover, surgical treatment was successful in all patients. 

### 4.4. Pathophysiology

A high prevalence of pHPT in patients with SCD (5%) in a previous study [11], a young age at onset, and a high rate of pHPT recurrence suggest that SCD may promote the development of pHPT. The first possible explanation involves vitamin D deficiency. Many reports have revealed that chronic vitamin D deficiency is highly prevalent and severe in adult and children patients with SCD and is associated with secondary hyperparathyroidism, which was not considered until recently and that SCD patients were not sufficiently supplemented throughout childhood and adulthood [11,24]. Several mechanisms can explain very low serum vitamin D concentrations in SCD patients, including low cutaneous synthesis, malabsorption of the liposoluble vitamin, and inflammatory conditions [11,24]. However, the relationship between vitamin D deficiency and pHPT remains unclear. Vitamin D deficiency is associated with more severe pHPT and may have a stimulatory effect on adenoma growth [29]. Vitamin D replacement has been shown to be safe and beneficial in patients with mild pHPT [30,31]. However, the hypothesis of chronic secondary hyperparathyroidism that could “autonomize” as an adenoma, as in tertiary hyperparathyroidism, in SCD patients remains speculative. 

Another pathophysiological explanation that could link pHPT and SCD is related to erythropoietin (EPO). Chronic hemolytic anemia associated with SCD induces high levels of EPO from the first months of life. Erythropoietin could chronically stimulate the parathyroid glands and may result in the development of parathyroid adenoma or hyperplasia over time. The presence of EPO receptors in the parathyroid gland in humans supports this hypothesis [32]. Moreover, one study found significantly increased serum PTH levels in healthy subjects after blood donation [33]. Nevertheless, the presence of four SC and two S/beta^+^thalassemia patients in our case series, genotypes generally associated with lower hemolytic parameters and higher hemoglobin level, contrasts with this hypothesis and pleads for other pathophysiological combined factors or difference in sensitivity to EPO levels between patients. It could be interesting to test this hypothesis in a mouse model and to study PTH hormone levels and parathyroid gland volumes in animals that are chronically stimulated with EPO, or with chronic anemia (phlebotomized mice or those with hemolytic anemia induced by phenylhydrazine).

A third mechanistic explanation could be a role of other growth factors on parathyroid adenoma formation. Indeed, plasma levels of angiogenesis and growth factors, such as vascular endothelial growth factor (VEGF) and fibroblastic growth factor (FGF), are increased in SCD compared to controls [34]. Interestingly, it was shown that these factors seem to play a role in human parathyroid adenoma cell proliferation in vitro, promoting parathyroid cells proliferation [35].

Otherwise, a question not resolved by our study is whether pHPT could affect the SCD activity, including the risk of vaso-occlusive crises. Human red cells contain Ca^2+^-activated K^+^ channels (Gardos channels) in their membranes. When the intracellular free Ca^2+^ concentration increases in red cells, the Gardos channel is activated and intracellular potassium is expelled along with water. The Gardos channel of sickle cells is known to play a major role in cell dehydration, resulting in sickling and vaso-occlusive crisis in SCD patients. A significant increase in potassium erythrocyte permeability has been reported in patients with hyperparathyroidism, which was reversible after parathyroidectomy [36], indicating that the Gardos effect may be increased in patients with hyperparathyroidism and could promote the occurrence of vaso-occlusive crisis in SCD patients. We observed a tendency toward a higher hemoglobin level and a lower reticulocyte rate after surgery (which was not statistically significant), which could support this hypothesis. In the two cases of SCD patients with pHPT reported in the literature [9,10], the frequency of painful episodes decreased after parathyroid surgery. Unfortunately, the number of vaso-occlusive crisis events was not properly recorded for our patients. A higher occurrence rate of vaso-occlusive crisis event in SCD patients with pHPT would be an additional reason to screen these patients for pHPT and to propose surgical treatment. 

The results of our study have several limitations, essentially due to its retrospective design. The small number of patients and missing data limit the strength of our results. Additionally, we were unable to estimate a reliable prevalence of pHPT in SCD patients. However, this is the first case series studying pHPT in adult patients with SCD. The association between pHPT and SCD needs to be confirmed in larger and prospective cohorts to assess its prevalence and impact on the course of SCD. 

Altogether, these data suggest to screen pHPT with an ionized calcemia in adult SCD patients (every two years, for example). If ionized calcemia (or total calcemia) is elevated, PTH level should be monitored. If PTH level is inappropriate (normal or elevated), a neck ultrasonography (US) and Tc99m-sestamibi scintigraphy will be required to localize parathyroid adenoma or hyperplasia. A BMD should be also performed to screen a low BMD. Parathyroidectomy should always be discussed in those young adults, even if this surgery is never urgent. Thus, it should be scheduled in a stable state of the SCD. In some frail SCD patients who cannot undergo surgery or in patients who are unwilling to do so, cinacalcet could be a medical option.

## 5. Conclusions

A young age at onset and a high rate of pHPT recurrence in patients with SCD suggest that pHPT could be an unknown metabolic complication of adult SCD. As pHPT is an additional risk factor for low BMD and fracture, pHPT screening with an ionized calcemia should be routinely performed in SCD patients, particularly those with low BMD. Since the life span of SCD patients is increasing, this complication could be more commonly observed in the future.

## Figures and Tables

**Table 1:** Clinical and radiological features of 28 adult patients with primary hyperparathyroidism and sickle cell disease at the time of pHPT diagnosis.

**Demographic parameters** Age at diagnosis (years)	41 (31.5–49.2)
Female	18 (64%)
Body mass index (kg/m^2^)	21.8 (19.4–25.1)
Homozygous (SS) sickle cell disease	22 (79%)
Sub-Saharan African origin	23 (82%)
**SCD treatment**	
Hydroxyurea *	15/27 (56%)
Median dosage (mg/day)	1000 (800–1500)
Exchange transfusion *	6/27 (22%)
Folic acid	27/27 (100%)
Vitamin D intake	22/27 (81%)
**pHPT clinical complication**	
Asymptomatic	16 (57%)
Kidney stone	3 (11%)
Gastric ulcer	2 (7%)
**Bone mineral density (*n* = 16)**	
Low BMD (< −2.5 < T-score < −1 SD)	2 (12.5%)
Very low BMD (≤−2.5 SD)	7 (44%)
Lumbar spine T-score	−1.1 (−3.05; +0.45)
Femoral neck T-score	−0.8 (−1.2; +2)
Distal radius T-score (*n* = 5)	−2.6 (−5.1; −1)

The values are expressed as the number of patients (%) or as the median (IQR); SCD, sickle cell disease; pHPT, primary hyperparathyroidism; SD, standard deviation. * Treatment duration: at least one year before pHPT diagnosis.

**Table 2:** Laboratory features of 28 adult patients with primary hyperparathyroidism and sickle cell disease at the time of pHPT diagnosis.

		n
Serum total calcium level (mmol/L)	2.62 (2.60–2.78)	28
Serum ionized calcium (mmol/L)	1.38 (1.35–1.42)	18
Serum phosphate level (mmol/L)	0.90 (0.81–0.99)	28
Hypophosphatemia *	8 (29%)	28
PTH (pg/mL)	105 (69–137)	27 **
Increased PTH level *	21 (78%)	27
Serum creatinine level (µmol/L)	58.5 (50.5–70.2)	28
eGFR (mL/min/1.73 m^2^)	110.5 (93.7–129.2)	28
25(OH)D (ng/mL)	25.9 (12.7–48.2)	24
1,25(OH)_2_D (pg/mL)	85 (53–121)	15
Hemoglobin (g/dL)	8.45 (7.7–10)	28
HbF (%)	8 (4–15)	24
MCV (fL)	91.5 (79.7–107.5)	28
Reticulocyte count (G/L)	200 (115–252)	27
Total bilirubin (µmol/L)	30.5 (19–43)	24
LDH (UI/L)	444 (305–716)	26
Neutrophil count (G/L)	3.8 (2.2–4.3)	28
CTX (nmol/L) (normal < 1.8)	4.3 (2.6–7.1)	12
Osteocalcin (ng/mL) (normal range 14–32)	35 (23–38)	13

The values are expressed in the number of patients (%) or as the median (IQR). PTH, serum parathyroid hormone concentration; eGFR, estimated glomerular filtration rate; 25(OH)D, serum 25-hydroxy-vitamin D concentration; 1,25 (OH)_2_D, serum; 1,25-dihydroxyvitamin D concentration; MCV, mean corpuscular volume; LDH, lactate dehydrogenase; CTX, C-terminal telopeptide of type I collagen. * Above or under the cut-off value of each center (the normal value ranges of each laboratory are detailed in Supplemental Table S1); ** PTH level was not retrospectively found in one patient who underwent surgery that confirmed a parathyroid adenoma.

**Table 3:** Comparison of bone mineral density (BMD) between SCD patients with pHPT and matched SCD controls without pHPT.

	Patients	Controls	*p*
	*n* = 16	*n* = 32	
Age	40 (34–49)	35 (31–43)	0.31
Female *	11 (69)	21 (66)	
Homozygous SCD	12 (75)	21 (66)	
Body mass index (kg/m^2^)	21.9 (19.6–25.8)	22.1 (20.3–25.2)	0.64
Hemoglobin (g/dl)	9 (7.7–10.7)	8.8 (7.5–10.5)	0.9
HbF (%)	7.8 (5–15)	8 (5.5–16)	0.8
Reticulocytes (G/L)	183 (88–240)	190 (100–250)	0.8
Neutrophil count (G/L)	3.6 (2.2–4.4)	4 (2.4–4.5)	0.7
Lumbar spine T-score	−1.1 (−3.05; +0.45)	−0.1 (−2.1; +0.72)	0.38
Femoral neck T-score	−0.8 (−1.2; +2)	0.4 (−0.65; +1.25)	0.68
Low BMD (<−2.5 < T-score < −1 SD)	2 (12.5)	10 (31)	0.073
Very low BMD (≤−2.5 SD)	7 (44)	4 (12.5)	0.027
25(OH)D (ng/mL)	26 (14–68)	15 (10–25)	0.05

The values are expressed as the number of patients (%) or as the median (IQR). SCD, sickle cell disease; 25(OH)D, serum 25-hydroxy-vitamin D concentration; SD, standard deviation. * Two were menopausal in each group.

**Table 4:** Laboratory features of 14 SCD patients with primary hyperparathyroidism and surgical management.

	Before Surgery	After Surgery	p
Calcemia (mmol/L)	2.65 (2.54–2.86)	2.23 (2.13–2.3)	0.0025
Phosphatemia (mmol/L)	0.89 (0.82–0.96)	1.2 (0.96–1.28)	0.056
PTH (pg/mL)	106 (93–145)	34 (31–50)	0.004
eGFR (ml/min/1.73 m^2^)	103 (91–131)	104 (85–124)	0.26
Hemoglobin (g/dL)	7.9 (6.75–9.7)	8.7 (7.8–9.6)	0.38
Reticulocyte count (G/L)	208 (135–285)	154 (122–237)	0.50

The values are expressed as the median (IQR). PTH, serum parathyroid hormone concentration; eGFR, estimated glomerular filtration rate.

**Table 5:** Comparison of SCD patients with primary hyperparathyroidism at the time of pHTP diagnosis according to their surgical management.

	Surgery (*n* = 14)	No Surgery (*n* = 14)	*p*
Age at diagnosis (years)	46 (30.5–51.5)	39 (34.2–44.7)	0.59
Female (%)	11 (79)	7 (50)	0.24
SCD genotype SS	12 (86)	10 (71)	0.65
Body mass index (kg/m^2^)	21.6 (19.5–23.7)	22.3 (19.5–26)	0.68
Low BMD (T-score < −1 SD)	6/9 (67)	2/7 (29)	0.66
Calcemia (mmol/L)	2.65 (2.54–2.86)	2.62 (2.6–2.71)	0.98
PTH (pg/mL)	106 (93–145)	88.5 (59.5–129.7)	0.19
Hemoglobin (g/dL)	7.9 (6.7–9.7)	9.2 (8.2–10.4)	0.09

The values are expressed as the median (IQR) or the number of patients (%); PTH, serum parathyroid hormone concentration.

## Supplementary Materials

The following are available online at https://www.mdpi.com/2077-0383/9/2/308/s1, Table S1: Laboratory normal value ranges in each hospital.
