# Supplementary material for: Primary Hyperparathyroidism in Sickle Cell Disease: An Unknown Complication of the Disease in Adulthood
Source: J Clin Med. 2020 Jan 22;9(2):308. doi: 10.3390/jcm9020308 (PMC7073651; doi:10.3390/jcm9020308)
Supplement: Supplementary file 1 [file jcm-09-00308-s001.pdf]

Supplementary Table

Laboratory normal value ranges in each hospital

	George Pompidou and Necker	Tenon	Mondor
Calcemia (mmol/L)	(2.23-2.58)	(2.16-2.52)	(2.15-2.55)
Serum ionized calcium (mmol/L)	(1.15-1.32)	(1.10-1.30)	(1.05-1.30)
Phosphatemia (mmol/L)	(0.87-1.46)	(0.85-1.31)	(0.80-1.45)
PTH (pg/mL)	(11-57)	(8-76)	(15-65)
